# Sub-Omohyoid Fascial Plane Block as a Diaphragm-Sparing Approach for Shoulder Analgesia: A Narrative Review

**DOI:** 10.3390/jcm15135178

**Published:** 2026-07-02

**Authors:** Siwook Chung, Dakyung Hong, Sung-woo Hyung, Hyeonsook Jee

**Affiliations:** Department of Anesthesiology and Pain Medicine, Eunpyeong St. Mary’s Hospital, College of Medicine, The Catholic University of Korea, Seoul 03312, Republic of Korea; swchung87@gmail.com (S.C.);

**Keywords:** sub-omohyoid fascial plane block, shoulder surgery, interscalene block, diaphragm-sparing block, regional anesthesia, brachial plexus, ultrasound-guided nerve block, postoperative analgesia, phrenic nerve, fascial plane block

## Abstract

Shoulder surgery often requires effective regional analgesia, but interscalene brachial plexus block, the current reference technique, is frequently associated with hemidiaphragmatic paresis and upper-extremity motor blockade. These limitations have driven interest in diaphragm-sparing approaches that preserve respiratory and functional recovery. The sub-omohyoid fascial plane block (SOFPB) is an emerging ultrasound-guided interfascial concept that targets the fascial compartment deep to the omohyoid muscle near the superior trunk–suprascapular nerve complex. In this narrative review, SOFPB is considered as an operational umbrella term for closely related sub-omohyoid approaches rather than as a fully standardized single technique. This review summarizes the anatomical rationale, sonoanatomy, proposed mechanisms, technical considerations, and available clinical evidence for SOFPB and related sub-omohyoid approaches in shoulder surgery. Current evidence suggests that sub-omohyoid approaches may provide clinically meaningful analgesia through suprascapular nerve involvement, partial superior trunk coverage, and variable interfascial spread while potentially reducing phrenic nerve exposure compared with conventional interscalene block. However, direct evidence remains limited and heterogeneous, with inconsistent nomenclature, variable injectate volumes, and insufficient standardized assessment of diaphragmatic function, motor preservation, and patient-centered outcomes. Cadaveric dye studies provide anatomical plausibility but should be interpreted as indirect and hypothesis-generating evidence rather than proof of clinical efficacy or respiratory safety. Therefore, SOFPB should currently be regarded as a promising but unvalidated addition to the spectrum of diaphragm-sparing shoulder blocks rather than a replacement for established techniques. Future anatomical imaging studies, dose-finding trials, and head-to-head randomized comparisons are needed to define its optimal role in contemporary shoulder regional anesthesia.

## 1. Introduction

### 1.1. Postoperative Pain After Shoulder Surgery

Shoulder surgery is frequently associated with moderate-to-severe postoperative pain, particularly during the early recovery period. Common procedures, including arthroscopic rotator cuff repair, shoulder stabilization surgery, fracture fixation, and shoulder arthroplasty, involve extensive manipulation of periarticular soft tissues, tendons, joint capsule, and osseous structures, resulting in substantial nociceptive stimulation. Inadequately controlled pain may impair sleep, delay mobilization, hinder rehabilitation, and reduce patient satisfaction.

Effective postoperative analgesia is especially important because pain control has implications beyond patient comfort. In a prospective observational study of arthroscopic shoulder surgery, Zeeni et al. demonstrated that postoperative pain intensity, opioid consumption, and early functional recovery were influenced by multiple perioperative factors, highlighting the importance of individualized pain management strategies in shoulder surgery [1]. Furthermore, persistent or severe pain during the early postoperative period may adversely affect functional outcomes. Guity et al. reported that increased early postoperative pain was associated with a higher incidence of postoperative shoulder stiffness following arthroscopic rotator cuff repair, emphasizing the importance of adequate analgesia to facilitate recovery and rehabilitation [2].

Pain management remains a major component of perioperative care across the spectrum of shoulder procedures, including shoulder arthroplasty. As highlighted in a recent review by Walker and Kamineni, contemporary postoperative pain management increasingly relies on multimodal analgesic strategies and regional anesthesia techniques to improve pain control while minimizing opioid-related adverse effects [3]. Consequently, optimizing perioperative analgesia has become a central objective in shoulder surgery, with growing interest in regional anesthesia techniques that provide effective pain relief while preserving motor and respiratory function.

### 1.2. Interscalene Block as the Current Gold Standard

Interscalene brachial plexus block (ISB) has long been regarded as the reference regional anesthesia technique for shoulder surgery because it provides dense, reliable analgesia for the shoulder joint and surrounding structures. By targeting the C5–C7 nerve roots and the upper trunk of the brachial plexus within the interscalene groove, ISB effectively anesthetizes the major neural pathways responsible for shoulder pain transmission [4].

Numerous clinical studies have demonstrated that ISB improves postoperative analgesia, reduces opioid consumption, and facilitates early recovery following a wide range of shoulder procedures, including arthroscopic rotator cuff repair, instability surgery, fracture fixation, and shoulder arthroplasty [3,4]. Consequently, ISB has become a central component of multimodal analgesic pathways and the benchmark against which emerging techniques are evaluated.

Despite its well-established analgesic benefits, increasing attention has been directed toward the adverse effects associated with ISB, particularly unintended phrenic nerve blockade and respiratory impairment. As highlighted by recent investigations into diaphragm-sparing alternatives, the search for techniques capable of preserving respiratory function while maintaining adequate analgesia has become a major focus of contemporary shoulder regional anesthesia research [5]. These concerns have prompted several alternative approaches, including superior trunk block and other selective techniques.

### 1.3. Limitations of Interscalene Block

Despite its well-established analgesic efficacy, ISB is associated with several limitations that may restrict its applicability in selected patient populations. Among these, ipsilateral phrenic nerve blockade resulting in hemidiaphragmatic paresis remains the most clinically significant concern. Because of the close anatomical relationship between the interscalene brachial plexus and the phrenic nerve, local anesthetic spread beyond the intended neural targets may compromise diaphragmatic function and reduce pulmonary reserve [4,5].

Although healthy individuals generally tolerate transient diaphragmatic dysfunction, the respiratory consequences may be clinically relevant in patients with pre-existing pulmonary disease, obesity, obstructive sleep apnea, advanced age, or contralateral diaphragmatic impairment. Efforts to mitigate this complication through modifications in injection technique or reductions in local anesthetic volume have shown variable success. Kim et al. demonstrated that hemidiaphragmatic paresis may still occur despite technical refinements designed to limit phrenic nerve involvement, suggesting that complete avoidance of diaphragmatic dysfunction remains challenging with conventional interscalene approaches [6].

In addition to respiratory impairment, ISB may produce undesirable effects such as upper-extremity motor blockade, Horner syndrome, recurrent laryngeal nerve palsy, and patient discomfort related to temporary motor weakness. These adverse effects have become increasingly important in the context of ambulatory surgery and enhanced recovery pathways, where the preservation of functional capacity and rapid postoperative recovery are prioritized.

Recent evidence has further highlighted the limitations of ISB by demonstrating that alternative brachial plexus approaches may provide comparable analgesia with reduced respiratory compromise. A systematic review and meta-analysis by Amaral et al. reported lower rates of hemidiaphragmatic paralysis and dyspnea with superior trunk block compared with ISB [7]. Similarly, a recent meta-analysis of randomized trials confirmed that diaphragmatic dysfunction remains a frequent consequence of brachial plexus blocks performed near the interscalene region and continues to be a major consideration in shoulder regional anesthesia [8]. Collectively, these findings have stimulated growing interest in diaphragm-sparing regional anesthesia techniques that seek to maintain effective analgesia while minimizing respiratory and motor impairment.

### 1.4. Emergence of Diaphragm-Sparing Alternatives

Growing recognition of the respiratory and motor limitations associated with ISB has stimulated the development of alternative regional anesthesia techniques for shoulder surgery. The primary objective of these approaches is to maintain effective postoperative analgesia while reducing unintended phrenic nerve blockade and preserving diaphragmatic function. Recent investigations have focused on more selective brachial plexus blockade, including superior trunk block, as well as combinations of suprascapular and axillary nerve blocks that target the principal sensory innervation of the shoulder while limiting proximal spread of local anesthetic [5,7].

Evidence accumulated over the past several years suggests that some of these diaphragm-sparing approaches may provide analgesic efficacy comparable to ISB while reducing the incidence of hemidiaphragmatic paralysis and respiratory symptoms [4,7,8]. Consequently, interest has expanded beyond conventional nerve-specific techniques toward novel interfascial plane blocks designed to achieve a more favorable balance between analgesic effectiveness and preservation of respiratory and motor function. Within this context, the sub-omohyoid fascial plane block (SOFPB) has recently emerged as a potential alternative strategy for perioperative analgesia in shoulder surgery.

### 1.5. Rationale, Definition, and Scope of SOFPB

The search for effective diaphragm-sparing analgesic techniques has led to increasing interest in regional anesthesia approaches targeting the sub-omohyoid region. Unlike conventional interscalene blockade, which relies on anesthetic deposition in close proximity to the brachial plexus roots and phrenic nerve, sub-omohyoid approaches seek to provide shoulder analgesia through a more distal and potentially selective anatomical pathway.

For the purpose of this review, the term “sub-omohyoid fascial plane block (SOFPB)” is used as an operational umbrella term rather than as a fully standardized single intervention. It refers to ultrasound-guided approaches in which local anesthetic is deposited in, or immediately adjacent to, the fascial compartment deep to the inferior belly of the omohyoid muscle and near the superior trunk–suprascapular nerve complex. This framework includes several closely related techniques reported under heterogeneous nomenclature, including subomohyoid suprascapular nerve block, subomohyoid anterior suprascapular block, infraclavicular subomohyoid block, and continuous subomohyoid suprascapular nerve blockade. These approaches share a sub-omohyoid anatomical corridor but differ in needle trajectory, intended primary target, injectate volume, and the degree to which they seek to block the suprascapular nerve, superior trunk, or a broader interfascial plane. Therefore, throughout this review, evidence derived from these techniques is interpreted as related but not always directly interchangeable with SOFPB as a distinct fascial plane concept. This definitional limitation is explicitly addressed in the terminology summary in Table 1 and in the limitations section.

Initial anatomical evidence supporting this concept was provided by Sehmbi et al., who demonstrated in a cadaveric dye study that injectate administered within the subomohyoid space could reach the suprascapular nerve and spread toward adjacent cervical structures [9]. Building upon these anatomical observations, Abdallah et al. subsequently evaluated the subomohyoid anterior suprascapular block in a multicenter randomized trial and demonstrated that clinically meaningful postoperative analgesia could be achieved following arthroscopic shoulder surgery using a sub-omohyoid approach [10]. Additional clinical experience has been reported with continuous subomohyoid suprascapular nerve blockade, suggesting the feasibility of prolonged postoperative analgesia in selected patients undergoing shoulder procedures [12].

Collectively, these findings support the hypothesis that the sub-omohyoid region may serve as a clinically relevant target for shoulder analgesia while potentially reducing the limitations associated with more proximal brachial plexus blockade. Although the precise mechanisms of action and patterns of injectate spread remain incompletely understood, growing anatomical and clinical evidence has established the foundation for the development of the sub-omohyoid fascial plane block (SOFPB). Consequently, SOFPB has emerged as a novel interfascial technique that warrants further investigation as a potential motor- and diaphragm-preserving alternative for perioperative analgesia in shoulder surgery.

### 1.6. Literature Search

This article is a focused narrative review based on a reproducible, non-systematic literature search. PubMed/MEDLINE was searched from database inception to 31 May 2026 for English-language publications using combinations of the following terms: “sub-omohyoid block,” “subomohyoid block,” “subomohyoid suprascapular nerve block,” “subomohyoid anterior suprascapular block,” “infraclavicular subomohyoid block,” “sub-omohyoid fascial plane block,” “anterior suprascapular nerve block,” “suprascapular nerve block,” “superior trunk block,” “upper trunk block,” “interscalene block,” “diaphragm-sparing,” “hemidiaphragmatic paresis,” “phrenic nerve,” “shoulder surgery,” and “postoperative analgesia.” Eligible records included randomized trials, comparative clinical studies, observational studies, case reports, cadaveric dye studies, anatomical investigations, systematic reviews, meta-analyses, and narrative reviews relevant to shoulder regional anesthesia or sub-omohyoid approaches. Reference lists of relevant articles were manually screened to identify additional sources. Because the aim was to synthesize anatomical, mechanistic, and clinical concepts rather than to generate a pooled quantitative estimate, no formal PRISMA flow diagram, risk-of-bias assessment, or meta-analysis was undertaken. The level and directness of the evidence were instead described narratively and summarized in the evidence tables.

## 2. Anatomical Basis of SOFPB

### 2.1. Omohyoid Muscle and the Sub-Omohyoid Space

A slender infrahyoid muscle, the omohyoid runs between the superior scapular border and the hyoid bone, with its superior and inferior bellies joined by an intermediate tendon. Along its oblique course through the lateral neck, it crosses several fascial compartments. Although traditionally associated with swallowing and stabilization of the hyoid apparatus, the omohyoid has recently gained attention as an important anatomical landmark in ultrasound-guided cervical procedures.

The inferior belly of the omohyoid courses through the posterior cervical triangle and crosses the supraclavicular region, where it lies in close proximity to the brachial plexus and adjacent neurovascular structures. Yang et al. demonstrated that the anatomical relationship between the omohyoid muscle and the internal jugular vein can be consistently identified using ultrasound, supporting the reliability of the omohyoid as a sonographic landmark in the lower cervical region [14]. These findings are particularly relevant for regional anesthesia because the omohyoid provides a readily recognizable reference structure for identifying the sub-omohyoid compartment.

The sub-omohyoid space represents a potential interfascial compartment located deep to the omohyoid muscle. Owing to its proximity to the suprascapular nerve origin, superior trunk region, and surrounding cervical fascial pathways, this compartment has emerged as a potential target for shoulder analgesia. Cadaveric evidence from Sehmbi et al. demonstrated that injectate deposited within the sub-omohyoid region may spread toward neural structures relevant to shoulder sensation, supporting the anatomical basis for sub-omohyoid regional anesthesia techniques [9].

Subsequent clinical investigations have further highlighted the importance of this anatomical region. Both single-injection and continuous subomohyoid approaches have been reported to provide effective postoperative analgesia following shoulder surgery [10,12]. Collectively, these findings suggest that the omohyoid is not merely a landmark but a structure defining the clinically relevant interfascial compartment underlying the sub-omohyoid fascial plane block.

### 2.2. Neural Pathways Involved in Shoulder Pain

The sensory innervation of the shoulder is complex and arises from multiple overlapping neural pathways. Pain originating from the glenohumeral joint, acromioclavicular joint, rotator cuff, subacromial bursa, and periarticular soft tissues is transmitted through a network of articular and periarticular branches derived from the brachial plexus and cervical plexus. This intricate innervation pattern partly explains the challenges of achieving complete shoulder analgesia using selective nerve blockade alone.

Recent anatomical studies have refined the understanding of shoulder sensory innervation. Laumonerie et al. proposed the concept of the “three bridges to break,” highlighting the major sensory contributions of the suprascapular, axillary, and lateral pectoral nerves to the shoulder joint [15]. Together, these nerves provide extensive articular innervation to the glenohumeral capsule and surrounding structures and represent key targets for contemporary regional anesthesia techniques.

Among these pathways, the suprascapular nerve is particularly important because it provides a substantial proportion of the sensory supply to the posterior and superior aspects of the shoulder joint. Originating from the superior trunk of the brachial plexus, the suprascapular nerve has, therefore, become a major target for several diaphragm-sparing analgesic approaches. Additional sensory input is provided by the axillary nerve, which supplies the inferior and posterolateral capsule, and by the lateral pectoral nerve, which contributes to anterior capsular innervation [15,16]. Contributions from the upper and lower subscapular nerves have also been described, further emphasizing the multifactorial nature of shoulder pain transmission [16].

Beyond the glenohumeral joint, cutaneous and periosteal sensation from the clavicular and superior shoulder regions may involve branches of the cervical plexus, particularly the supraclavicular nerves. Cadaveric evidence has demonstrated variable innervation of the clavicle from both cervical plexus and brachial plexus-derived branches, suggesting that clavicular and peri-incisional pain may not be fully addressed by isolated blockade of the major articular nerves [17].

This anatomical complexity has important implications for SOFPB. Rather than relying on the blockade of a single terminal nerve, the proposed mechanism of SOFPB may involve modulation of multiple neural pathways located near the superior trunk and suprascapular nerve origin. Consequently, understanding the relationship between the sub-omohyoid compartment and these neural structures is essential for interpreting the potential analgesic effects of this technique.

### 2.3. Anatomical Relationship Between the Sub-Omohyoid Space and the Brachial Plexus

The anatomical rationale for SOFPB is largely based on the close relationship between the sub-omohyoid compartment and the proximal brachial plexus. The superior trunk is formed by the convergence of the C5 and C6 nerve roots and gives rise to the suprascapular nerve, one of the principal sensory contributors to the shoulder joint. As the suprascapular nerve courses laterally toward the suprascapular notch, it traverses the lower cervical region in proximity to the inferior belly of the omohyoid muscle and adjacent fascial planes. This anatomical arrangement creates a potential pathway through which local anesthetic deposited beneath the omohyoid muscle may influence neural structures involved in shoulder pain transmission [15,16].

Cadaveric investigations have provided important insights into this relationship. In a dye study of ultrasound-guided subomohyoid suprascapular nerve block, Sehmbi et al. demonstrated that injectate deposited within the sub-omohyoid region could reach the suprascapular nerve while spreading through adjacent cervical fascial planes [9]. These findings supported the concept that the sub-omohyoid compartment may serve as an anatomical conduit linking the site of injection to sensory pathways relevant to shoulder analgesia. Building upon this anatomical foundation, Abdallah et al. subsequently evaluated the subomohyoid anterior suprascapular block in a multicenter randomized trial, providing clinical evidence that a sub-omohyoid approach can achieve effective postoperative analgesia following arthroscopic shoulder surgery [10].

Recent anatomical evidence has further refined the understanding of injectate spread in this region. Diwan et al. demonstrated that dye injected through an anterior suprascapular nerve block approach could spread posterior to the brachial plexus and, in some specimens, extend toward structures associated with the phrenic nerve [13]. These findings suggest that the clinical effects of sub-omohyoid approaches may not be explained solely by blockade of the suprascapular nerve. Instead, local anesthetic spread may involve multiple interconnected fascial pathways surrounding the superior trunk and adjacent neural structures.

Therefore, the sub-omohyoid space should be regarded not as an isolated anatomical compartment but as a strategically positioned interfascial region located near the superior trunk–suprascapular nerve complex. This relationship provides a plausible anatomical basis for the analgesic effects observed with SOFPB while also highlighting the need for further imaging and cadaveric studies to clarify its precise mechanism of action and patterns of injectate distribution. In contrast to an interscalene block, which targets the brachial plexus roots close to the phrenic nerve, SOFPB is performed at a more distal location; its analgesic effect is therefore likely mediated by a combination of suprascapular nerve involvement, partial superior trunk coverage, and interfascial spread within the lower cervical region.

## 3. Technical Considerations and Proposed Mechanisms

**Figure 1:**
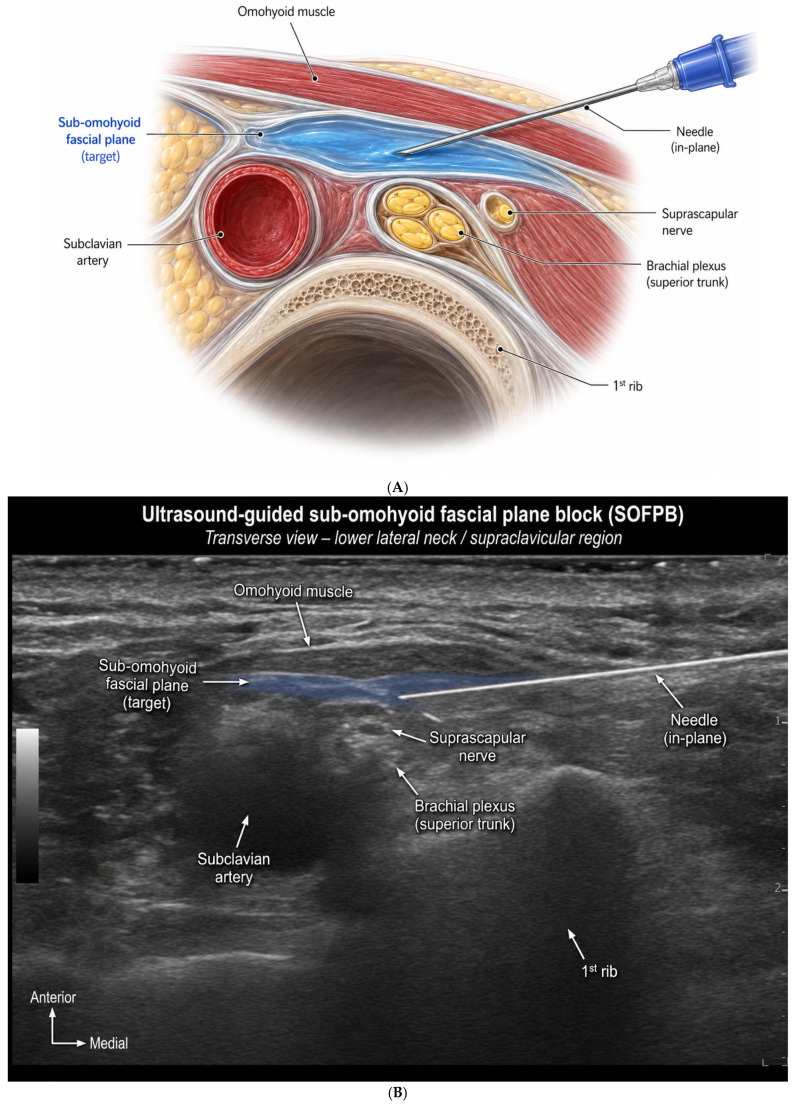
(**A**) Anatomical schematic corresponding to the sonographic view shown in (**B**), illustrating the omohyoid muscle, subclavian artery, brachial plexus (superior trunk), suprascapular nerve, first rib, in-plane needle trajectory, and the sub-omohyoid fascial plane target for local anesthetic deposition. Schematic illustration, not to scale. (**B**) Representative annotated ultrasound image of the lower lateral neck/supraclavicular region demonstrating the omohyoid muscle, subclavian artery, brachial plexus (superior trunk region), suprascapular nerve, in-plane needle trajectory, and sub-omohyoid fascial plane target. The needle and local anesthetic spread are shown as schematic overlays to emphasize interfascial rather than intraneural deposition.

Accurate identification of the sub-omohyoid compartment and adjacent neural structures is fundamental to the safe and effective performance of SOFPB. The target region is located within the lower cervical area, where the inferior belly of the omohyoid muscle serves as a key sonographic landmark because of its consistent anatomical relationship with adjacent neurovascular structures. To improve practical reproducibility for perioperative physicians, an annotated ultrasound image of the relevant sonoanatomy is provided together with the schematic anatomy (Figure 1B).

Once the omohyoid muscle is identified, the transducer is adjusted to define the sub-omohyoid plane and the deeper neurovascular landmarks, including the subclavian artery and the brachial plexus elements near the superior trunk and suprascapular nerve region. The proposed sub-omohyoid target plane is located deep to the omohyoid muscle and adjacent to, but not within, the superior trunk–suprascapular nerve complex. In clinical practice, the needle should be advanced under continuous in-plane ultrasound visualization, and correct placement is suggested by separation of the fascial plane rather than by direct needle-to-nerve contact.

Successful identification of the omohyoid muscle, surrounding fascial layers, and the underlying brachial plexus is fundamental to the accurate performance of SOFPB and may influence both analgesic efficacy and unintended spread to adjacent structures. Inadequate visualization may increase the risk of vascular puncture, neural injury, or unintended spread to non-target structures, including the phrenic nerve.

### 3.1. Block Technique

Although technical variations have been described, SOFPB is generally performed under ultrasound guidance with the patient in the supine or semi-sitting position and the head rotated contralaterally. A high-frequency linear transducer is placed over the lower lateral neck to identify the sternocleidomastoid muscle, internal jugular vein, carotid artery, omohyoid muscle, and deeper brachial plexus structures. Particular attention is directed toward identifying the inferior belly of the omohyoid muscle, which serves as the principal sonographic landmark for localization of the target compartment [14].

After visualization of the relevant anatomy, the transducer is adjusted to optimize the relationship between the omohyoid muscle and the superior trunk region of the brachial plexus. The target injection plane is typically located deep to the omohyoid muscle and superficial to the deeper cervical structures adjacent to the superior trunk–suprascapular nerve complex. Most operators advance the needle in plane, keeping the tip under continuous view as it is directed into the sub-omohyoid compartment [9,10].

Once aspiration has excluded intravascular placement, local anesthetic is delivered in small increments, with fascial plane separation and subomohyoid spread observed in real time. Real-time ultrasound visualization is essential because the effectiveness of SOFPB is believed to depend on appropriate interfascial distribution rather than direct needle-to-nerve contact. Consequently, confirmation of injectate spread within the intended compartment may be more important than precise localization of an individual neural structure.

Technical variations and nomenclature among published sub-omohyoid approaches are summarized in Table 2. This table is intended to clarify that SOFPB is used here as an operational framework for anatomically related techniques rather than as a universally standardized block.

### 3.2. Injectate Volume and Spread Pattern

The clinical effects of SOFPB are believed to depend largely on the distribution of local anesthetic within the sub-omohyoid compartment rather than direct deposition around a single neural target. Consequently, injectate volume and spread pattern are likely important determinants of both analgesic efficacy and unintended neural involvement.

Published studies have used varying approaches and local anesthetic volumes, reflecting the absence of a standardized technique. In general, the objective is to achieve adequate interfascial spread beneath the omohyoid muscle and around the superior trunk–suprascapular nerve complex while minimizing extension toward non-target structures. Because SOFPB is performed within a fascial plane rather than immediately adjacent to an isolated nerve, the final distribution of local anesthetic may be influenced by fascial continuity, tissue compliance, injection pressure, and total injectate volume.

Cadaveric investigations provide important insights into potential spread patterns. Sehmbi et al. demonstrated that dye injected within the subomohyoid region could reach the suprascapular nerve through the surrounding fascial planes, supporting the anatomical basis for shoulder analgesia following sub-omohyoid approaches [9]. More recently, Diwan et al. reported that injectate administered through an anterior suprascapular nerve block approach could spread posterior to the brachial plexus and, in some specimens, extend toward structures associated with the phrenic nerve [13]. These findings suggest that injectate distribution may not be limited to the suprascapular nerve alone and may vary according to anatomical characteristics and injection technique.

Given the limited anatomical and clinical evidence currently available, future studies incorporating imaging modalities such as MRI or CT contrast studies and standardized volume protocols are needed to better define the spread characteristics of SOFPB and their relationship to clinical outcomes. Importantly, cadaveric dye studies provide anatomical plausibility rather than direct evidence of clinical analgesic efficacy or respiratory safety. Findings from such studies should therefore be interpreted as hypothesis-generating and cannot be assumed to predict in vivo local anesthetic behavior, diaphragmatic function, or patient-centered outcomes.

Given the limited anatomical and clinical evidence currently available, future studies incorporating imaging modalities such as MRI or CT contrast studies and standardized volume protocols are needed to better define the spread characteristics of SOFPB and their relationship to clinical outcomes.

### 3.3. Proposed Mechanisms of Analgesia

The precise mechanism of analgesia following SOFPB remains incompletely understood. Unlike conventional peripheral nerve blocks that target a discrete neural structure, SOFPB is an interfascial technique in which local anesthetic is deposited within the sub-omohyoid compartment. Consequently, its analgesic effects are likely mediated through a combination of neural blockade and fascial spread rather than inhibition of a single nerve.

The most plausible mechanism involves the suprascapular nerve, which originates from the superior trunk of the brachial plexus and provides a substantial proportion of the sensory innervation to the glenohumeral and acromioclavicular joints [15,16]. Cadaveric evidence has demonstrated that injectate introduced into the sub-omohyoid region can reach the suprascapular nerve, supporting the hypothesis that suprascapular nerve blockade represents a major contributor to the analgesic effects of SOFPB [9]. This concept is consistent with growing evidence supporting anterior suprascapular nerve block (ASSB) as an effective analgesic strategy for shoulder surgery. Randomized controlled trials and comparative studies have demonstrated that ASSB provides clinically meaningful postoperative analgesia while reducing respiratory impairment compared with interscalene block [18,19,20]. Furthermore, a systematic review and meta-analysis by White et al. confirmed the analgesic efficacy of anterior suprascapular approaches for arthroscopic shoulder surgery [21].

A second potential mechanism involves partial blockade of the superior trunk. Because the sub-omohyoid compartment lies adjacent to the superior trunk–suprascapular nerve complex, local anesthetic may spread beyond the suprascapular nerve and affect neighboring C5–C6 sensory fibers. Such spread may enhance analgesic coverage by influencing multiple neural pathways involved in shoulder nociception, including contributions from the axillary and lateral pectoral nerves [15,16].

Taken together, current evidence supports a plausible but still unproven multifactorial mechanism involving suprascapular nerve blockade, partial superior trunk coverage, and interfascial spread within the lower cervical compartment. The relative contribution of each pathway remains uncertain, and mechanistic inferences from anatomical and dye-diffusion studies should not be interpreted as direct proof of clinical efficacy.

Taken together, current evidence supports a multifactorial mechanism involving suprascapular nerve blockade, partial superior trunk coverage, and interfascial spread within the lower cervical compartment. Although the relative contribution of each pathway remains uncertain, this combined mechanism may explain the favorable analgesic outcomes reported with sub-omohyoid approaches while preserving a potentially more selective block profile than conventional interscalene techniques.

### 3.4. Potential Diaphragm- and Motor-Sparing Characteristics

The proposed diaphragm-sparing characteristics of SOFPB are primarily derived from its anatomical location and presumed mechanism of action. Unlike ISB, which targets the brachial plexus roots within the interscalene groove, SOFPB is performed in the sub-omohyoid compartment adjacent to the superior trunk–suprascapular nerve complex. This more distal target is anatomically positioned to potentially reduce phrenic nerve exposure, although this has not been confirmed in adequately powered comparative trials specifically evaluating SOFPB. Similar principles have been demonstrated in anterior suprascapular nerve block (ASSB), which has consistently shown lower rates of diaphragmatic dysfunction than ISB while providing clinically acceptable postoperative analgesia in randomized clinical trials [18,19,20,21].

The rationale for selective shoulder analgesia is further supported by systematic reviews demonstrating that suprascapular nerve-based approaches can provide effective pain control with fewer block-related adverse effects than conventional interscalene techniques [21,22]. These findings have stimulated growing interest in distal and interfascial approaches, including SOFPB, as potential alternatives for patients at increased risk of respiratory complications.

Nevertheless, current evidence does not support the assumption that phrenic nerve involvement can be completely eliminated. Cadaveric investigations have demonstrated that injectate administered through anterior suprascapular approaches may spread beyond the intended target and reach structures associated with the phrenic nerve [13]. Furthermore, studies of ISB have shown that respiratory effects are influenced not only by anatomical target selection but also by injectate volume and spread characteristics [23]. Given the interfascial nature of SOFPB, similar factors may contribute to variability in diaphragmatic function and clinical outcomes.

Potential preservation of upper-extremity motor function represents another theoretical advantage of SOFPB. Because the technique may preferentially influence sensory pathways without extensive brachial plexus blockade, it could reduce postoperative motor weakness compared with ISB. However, objective evidence regarding motor preservation remains limited. At present, SOFPB should be regarded as a promising diaphragm- and motor-sparing strategy whose clinical benefits require confirmation through prospective studies incorporating standardized assessments of respiratory and motor function.

## 4. Clinical Evidence

### 4.1. Clinical Studies of SOFPB

Clinical evidence supporting SOFPB remains limited compared with established shoulder analgesic techniques such as interscalene brachial plexus block (ISB) and suprascapular nerve block. Nevertheless, the available literature provides preliminary insights into the feasibility, analgesic efficacy, and respiratory profile of sub-omohyoid approaches.

Interest in the sub-omohyoid region as a target for shoulder analgesia was initially stimulated by the report of Sondekoppam et al., who proposed the subscapularis and sub-omohyoid plane blocks as alternatives to conventional peripheral nerve blocks for shoulder surgery [24]. Although this report was primarily conceptual, it established the anatomical basis for subsequent investigations of sub-omohyoid techniques.

The first prospective clinical evidence was provided by Taha et al., who compared an infraclavicular subomohyoid block with a low-volume ISB in a randomized blinded study [11]. The authors demonstrated a lower incidence of diaphragmatic dysfunction while maintaining satisfactory analgesic outcomes, suggesting that targeting the sub-omohyoid compartment may offer a respiratory advantage compared with conventional interscalene approaches. This study provided early clinical support for the concept of diaphragm-sparing shoulder analgesia.

Subsequent investigations focused on refining anterior suprascapular and sub-omohyoid approaches. Abdallah et al. conducted a multicenter randomized trial comparing a subomohyoid anterior suprascapular block with ISB for arthroscopic shoulder surgery [10]. The study demonstrated clinically effective postoperative analgesia while supporting the feasibility of a more selective shoulder block strategy. Although ISB was superior for some analgesic outcomes, the trial supported sub-omohyoid approaches as a reasonable alternative when respiratory preservation is a priority.

Additional evidence remains limited. Dhir et al. reported successful use of continuous bilateral subomohyoid suprascapular nerve blocks for postoperative analgesia following bilateral rotator cuff repair, demonstrating the feasibility of catheter-based techniques within the sub-omohyoid compartment [12]. Furthermore, cadaveric investigations by Sehmbi et al. and Diwan et al. provided anatomical evidence supporting injectate spread toward the suprascapular nerve and adjacent cervical structures, thereby offering mechanistic explanations for the clinical observations reported in early studies [9,13].

As summarized in Table 3, the current evidence base for SOFPB and related sub-omohyoid approaches remains relatively limited and consists of a mixture of anatomical studies, early clinical investigations, and a small number of comparative trials. To help readers judge robustness, the table distinguishes clinical comparative evidence from cadaveric or feasibility evidence and summarizes available quantitative findings or effect directions where reported.

### 4.2. SOFPB in the Context of Contemporary Diaphragm-Sparing Shoulder Blocks

**Figure 2:**
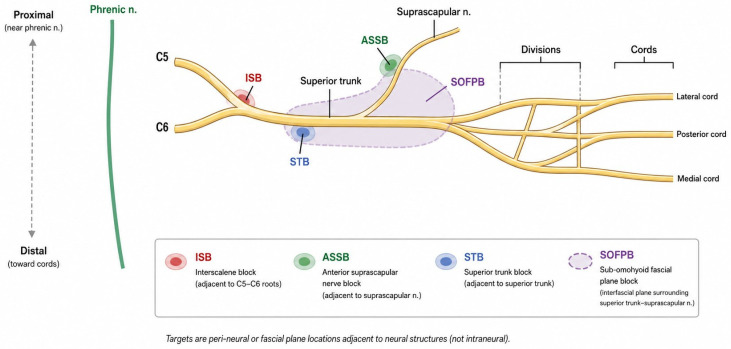
Relative injection sites of diaphragm-sparing shoulder blocks along the brachial plexus. More proximal deposition lies closer to the phrenic nerve and carries a higher risk of hemidiaphragmatic paresis, whereas more distal and selective approaches—including the sub-omohyoid fascial plane block—progressively reduce this risk. Schematic illustration, not to scale.

Increasing recognition of the respiratory limitations associated with interscalene brachial plexus block (ISB) has driven the development of alternative regional anesthesia techniques that seek to preserve diaphragmatic function while maintaining effective postoperative analgesia. Consequently, contemporary shoulder regional anesthesia has progressively shifted from complete brachial plexus blockade toward more selective and anatomically targeted approaches. Within this evolving paradigm, SOFPB should be considered as part of a broader family of diaphragm-sparing shoulder blocks rather than as an isolated technique (Figure 2).

**Figure 3:**
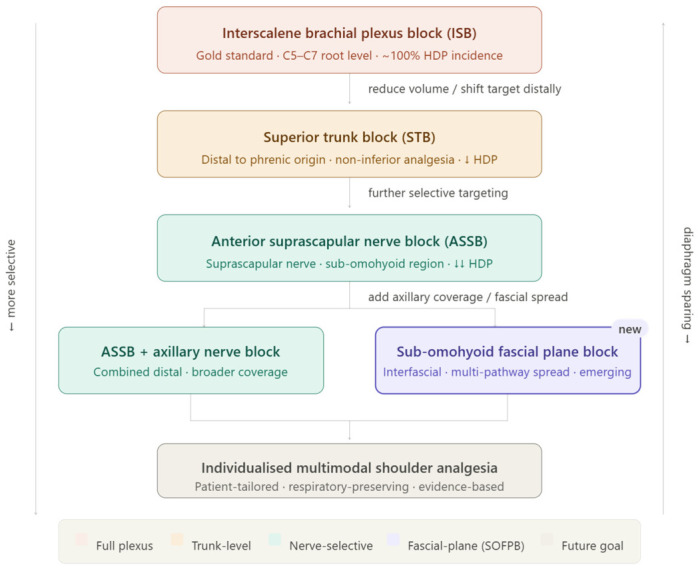
Evidence hierarchy and evolution of diaphragm-sparing shoulder blocks, with SOFPB positioned as an emerging interfascial approach.

Among currently available alternatives, anterior suprascapular nerve block (ASSB) has become one of the most extensively investigated strategies. Because the suprascapular nerve contributes substantially to sensory innervation of the glenohumeral joint, selective blockade of this nerve may provide meaningful analgesia while minimizing unintended blockade of adjacent neural structures. Multiple randomized clinical trials have demonstrated lower rates of diaphragmatic dysfunction with ASSB compared with ISB while maintaining clinically acceptable postoperative analgesia [18,19,20] (Figure 3). These findings are further supported by systematic reviews and meta-analyses demonstrating that suprascapular nerve-based approaches provide effective pain control while reducing some of the respiratory consequences associated with conventional interscalene techniques [21,22,25].

Recent investigations have focused not only on efficacy but also on optimization of injectate volume. Dose-finding studies by Coşarcan et al. and Pu et al. established effective local anesthetic volumes for ultrasound-guided ASSB, highlighting the importance of balancing adequate analgesia against excessive injectate spread to adjacent neural structures [26,27]. These observations are particularly relevant to SOFPB because injectate distribution within cervical fascial compartments is believed to play a major role in determining both analgesic efficacy and unintended phrenic nerve involvement.

Another major development has been the emergence of superior trunk block (STB) and related upper trunk approaches. By targeting the brachial plexus distal to the nerve roots while maintaining coverage of the C5–C6 distribution, STB seeks to preserve analgesic efficacy while reducing phrenic nerve dysfunction. Lee et al. demonstrated that STB provided anesthetic quality comparable to ISB in arthroscopic shoulder surgery [28]. Similarly, Kang et al. reported noninferior postoperative analgesia with STB compared with conventional interscalene block [29]. Further refinement of selective proximal brachial plexus techniques has been explored through upper trunk approaches; Zhang et al. demonstrated that a subparaneural upper trunk block achieved noninferior analgesia compared with ISB, supporting the concept that effective shoulder analgesia may be obtained without routine blockade of the entire interscalene compartment [30]. More recently, a systematic review and meta-analysis by Amaral et al. concluded that STB represents an effective phrenic-sparing alternative to ISB for shoulder arthroscopy [7].

Within this spectrum of contemporary diaphragm-sparing techniques, SOFPB occupies a provisional and still incompletely defined position. Unlike ASSB, STB, or upper trunk block, which primarily target discrete neural structures, SOFPB is conceptualized as an interfascial approach within the sub-omohyoid compartment. This strategy may facilitate spread toward the suprascapular nerve, superior trunk, and adjacent cervical fascial pathways simultaneously, but the consistency and clinical significance of this spread remain uncertain. Direct comparative evidence is currently insufficient to conclude that SOFPB is superior to established diaphragm-sparing alternatives.

Within this spectrum of contemporary diaphragm-sparing techniques, SOFPB occupies a unique position. Unlike ASSB, STB, or upper trunk block, which primarily target discrete neural structures, SOFPB utilizes an interfascial approach within the sub-omohyoid compartment. This strategy may facilitate spread toward the suprascapular nerve, superior trunk, and adjacent cervical fascial pathways simultaneously, potentially providing broader sensory coverage while retaining some of the respiratory advantages associated with more selective shoulder blocks. However, direct comparative evidence remains limited.

Despite increasing interest in diaphragm-sparing regional anesthesia, no single technique has yet emerged as the definitive alternative to ISB. Existing studies remain heterogeneous with respect to injectate volume, block technique, outcome assessment, and definitions of respiratory dysfunction. Consequently, the precise role of SOFPB relative to ASSB, STB, upper trunk approaches, and combined distal block strategies remains incompletely defined (Figure 2, Table 4).

### 4.3. Analgesic Efficacy

The primary objective of any shoulder regional anesthesia technique is to provide effective postoperative analgesia while minimizing procedure-related adverse effects. Although direct clinical evidence supporting SOFPB remains limited, currently available studies suggest that sub-omohyoid approaches may provide clinically meaningful postoperative pain relief and should be interpreted within the broader context of contemporary shoulder regional anesthesia literature.

Direct evidence for the analgesic efficacy of SOFPB is primarily derived from the multicenter randomized trial conducted by Abdallah et al., which compared a subomohyoid anterior suprascapular block with interscalene brachial plexus block (ISB) in patients undergoing arthroscopic shoulder surgery [10]. While ISB demonstrated superior analgesic performance for certain postoperative outcomes, the subomohyoid approach provided satisfactory pain control and supported the feasibility of achieving effective shoulder analgesia through a more selective regional anesthesia strategy. These findings suggest that complete blockade of the brachial plexus may not be necessary to obtain clinically acceptable postoperative analgesia.

Evidence from anterior suprascapular nerve block (ASSB) studies further supports this concept. Randomized controlled trials have consistently demonstrated effective postoperative analgesia with ASSB while simultaneously reducing respiratory impairment compared with ISB [19,20]. Furthermore, systematic reviews and meta-analyses have shown that suprascapular nerve-based techniques provide clinically meaningful pain relief after arthroscopic shoulder surgery, highlighting the importance of the suprascapular pathway as a target for perioperative analgesia [21,31,32,33].

More recently, network meta-analyses have expanded the comparative evidence base by evaluating multiple regional anesthesia techniques simultaneously. Liu et al. demonstrated that analgesic efficacy varies among shoulder block techniques, although several diaphragm-sparing approaches achieved favorable postoperative pain outcomes [25]. Similar conclusions were reported by Hurley et al. and Wu et al., who identified substantial differences among regional analgesic strategies while emphasizing the overall effectiveness of peripheral nerve blocks in reducing postoperative pain following shoulder arthroscopy [39,40]. In addition, Kalthoff et al. demonstrated that peripheral nerve block techniques consistently outperform general anesthesia alone for pain control after arthroscopic rotator cuff repair, reinforcing the central role of regional anesthesia in contemporary shoulder surgery [41].

Taken together, current evidence indicates that effective postoperative analgesia can be achieved through a variety of selective neural and interfascial approaches. Although direct clinical data regarding SOFPB remain limited, available findings are consistent with the broader literature supporting suprascapular nerve-based and diaphragm-sparing shoulder blocks. These findings should be interpreted as supportive but indirect rather than as definitive evidence that SOFPB is equivalent or superior to established techniques.

Taken together, current evidence indicates that effective postoperative analgesia can be achieved through a variety of selective neural and interfascial approaches. Although direct clinical data regarding SOFPB remain limited, available findings are consistent with the growing body of literature supporting suprascapular nerve-based and diaphragm-sparing shoulder blocks.

### 4.4. Respiratory and Functional Outcomes

Preservation of respiratory function has become a central objective in the development of contemporary shoulder regional anesthesia techniques. Although interscalene brachial plexus block (ISB) remains the benchmark for postoperative analgesia, its close anatomical relationship with the phrenic nerve frequently results in ipsilateral hemidiaphragmatic paresis. While this consequence is often clinically insignificant in healthy individuals, it may adversely affect patients with obesity, advanced age, pulmonary disease, or limited respiratory reserve [42]. Consequently, considerable efforts have been directed toward identifying alternative techniques that maintain analgesic efficacy while minimizing respiratory compromise.

The influence of injectate volume on phrenic nerve involvement has long been recognized. In a landmark randomized study, Riazi et al. demonstrated that reducing local anesthetic volume during ultrasound-guided ISB significantly decreased respiratory impairment while preserving analgesic effectiveness [23]. However, subsequent investigations have confirmed that even low-volume ISB does not completely eliminate the risk of hemidiaphragmatic paresis, prompting continued exploration of more selective regional anesthesia strategies [42,43].

Among these alternatives, anterior suprascapular nerve block (ASSB) has consistently demonstrated favorable respiratory outcomes. Lim et al. reported superior preservation of diaphragmatic function following ASSB compared with ISB while maintaining clinically acceptable postoperative analgesia [19]. Similar findings were subsequently reported by Doğan et al. and Gupta et al., both of whom observed lower rates of diaphragmatic dysfunction after ASSB than after conventional interscalene blockade [18,20]. Collectively, these studies support the concept that selective blockade of shoulder sensory pathways can reduce unintended phrenic nerve involvement.

Comparable observations have emerged from the superior trunk block (STB) literature. Randomized trials by Kang et al., Lee et al., and Zhang et al. demonstrated that effective shoulder analgesia can be achieved through more distal brachial plexus targets while potentially reducing respiratory compromise compared with ISB [28,29,30]. A subsequent systematic review and meta-analysis by Amaral et al. further supported STB as an effective phrenic-sparing alternative for shoulder arthroscopy [7]. More recent investigations have focused on optimizing injectate volume within STB. Wang et al. demonstrated that low-volume ropivacaine administration improved preservation of diaphragmatic movement after shoulder arthroscopy [34], while a subsequent dose-finding study identified local anesthetic volumes associated with a high probability of avoiding hemidiaphragmatic paralysis [35]. These findings reinforce the importance of injectate volume as a modifiable determinant of respiratory outcomes.

The respiratory implications of sub-omohyoid approaches remain less well defined. Taha et al. demonstrated reduced diaphragmatic impairment with an infraclavicular subomohyoid technique compared with low-volume ISB, providing preliminary clinical evidence supporting a diaphragm-sparing effect [11]. However, cadaveric and anatomical investigations suggest that injectate spread toward structures associated with the phrenic nerve remains possible under certain conditions, indicating that respiratory preservation cannot be assumed universally [13].

Overall, available evidence suggests that diaphragm-sparing shoulder blocks can substantially reduce respiratory impairment while maintaining clinically effective analgesia. Although preliminary findings support potential respiratory advantages of sub-omohyoid approaches, the true incidence of hemidiaphragmatic paresis after SOFPB remains unknown. Future studies incorporating standardized diaphragmatic ultrasound assessment, pulmonary function testing, and patient-centered functional outcomes are required to define its role among contemporary shoulder regional anesthesia techniques.

Overall, available evidence suggests that diaphragm-sparing shoulder blocks can substantially reduce respiratory impairment while maintaining clinically effective analgesia. Although preliminary findings support potential respiratory advantages of SOFPB, direct comparative evidence remains limited. Future studies incorporating standardized diaphragmatic ultrasound assessment, pulmonary function testing, and patient-centered functional outcomes are required to define its role among contemporary shoulder regional anesthesia techniques.

Despite increasing interest in SOFPB as a potential diaphragm-sparing alternative for shoulder analgesia, the current body of evidence remains limited, and several important gaps must be acknowledged. First, direct clinical evidence supporting SOFPB is sparse. Compared with established techniques such as interscalene brachial plexus block (ISB), anterior suprascapular nerve block (ASSB), and superior trunk block (STB), only a small number of clinical studies have evaluated sub-omohyoid approaches [10,11,44]. Moreover, these studies do not always examine the same anatomical target or technical endpoint. Consequently, many proposed advantages of SOFPB rely on extrapolation from related techniques and anatomical plausibility rather than robust direct comparative clinical evidence.

Despite increasing interest in SOFPB as a potential diaphragm-sparing alternative for shoulder analgesia, the current body of evidence remains limited, and several important gaps must be acknowledged. First, direct clinical evidence supporting SOFPB is relatively sparse. Compared with established techniques such as interscalene brachial plexus block (ISB), anterior suprascapular nerve block (ASSB), and superior trunk block (STB), only a small number of clinical studies have specifically evaluated sub-omohyoid approaches [10,11,44]. Consequently, many proposed advantages of SOFPB continue to rely on anatomical rationale rather than robust comparative clinical evidence.

Third, terminology remains inconsistent. The terms “sub-omohyoid fascial plane block,” “subomohyoid anterior suprascapular block,” “subomohyoid suprascapular nerve block,” and “infraclavicular subomohyoid block” are not used uniformly in the literature. As a result, the present review uses SOFPB as an operational umbrella term while acknowledging that these approaches are related but not necessarily identical. This lack of standardized nomenclature creates challenges when comparing anatomical, cadaveric, and clinical studies and may partially explain discrepancies in reported injectate spread patterns and clinical outcomes [10,13].

Third, terminology remains inconsistent. The terms “sub-omohyoid fascial plane block,” “subomohyoid anterior suprascapular block,” and related descriptions are not always used uniformly in the literature. This lack of standardized nomenclature creates challenges when comparing anatomical, cadaveric, and clinical studies and may partially explain discrepancies in reported injectate spread patterns and clinical outcomes [10,13].

Another important limitation is the lack of standardized outcome assessment. Although diaphragm-sparing characteristics are frequently emphasized, studies vary considerably in their methods for evaluating respiratory function. Ultrasound assessment of diaphragmatic excursion, pulmonary function testing, and clinical measures of respiratory impairment are not consistently applied across investigations [18,19,20,45,46]. Similarly, patient-centered outcomes such as functional recovery, upper-extremity motor preservation, quality of recovery, and return to daily activities remain underreported.

Finally, direct head-to-head comparisons between SOFPB and contemporary diaphragm-sparing alternatives remain scarce. While the evidence base supporting ASSB and STB has expanded substantially through randomized trials and meta-analyses [7,21,25,29,31,32,33,39,40,41], comparable data for SOFPB are lacking. Future research should prioritize standardized terminology, imaging-confirmed injectate spread analysis, objective respiratory assessment, and adequately powered comparative trials to better define the role of SOFPB within modern shoulder regional anesthesia.

## 5. Discussion

### 5.1. Positioning SOFPB Within the Evolution of Shoulder Regional Anesthesia

The evolution of shoulder regional anesthesia over the past decade has been characterized by a progressive shift from comprehensive neural blockade toward increasingly selective and function-preserving approaches. Historically, interscalene brachial plexus block (ISB) has been regarded as the gold standard for shoulder analgesia because of its reliable blockade of the C5–C6 nerve roots and consistently favorable analgesic outcomes. However, the near-universal occurrence of ipsilateral hemidiaphragmatic paresis and the potential for upper-extremity motor impairment have prompted the development of alternative techniques designed to preserve respiratory and functional capacity while maintaining effective analgesia [42,43].

This transition has led to the emergence of several diaphragm-sparing strategies. As detailed in Section 4.2, anterior suprascapular nerve block (ASSB) [18,19,20,21,25,31,32,33] and superior or upper trunk approaches [7,28,29,30,34,35,36] were each introduced to preserve diaphragmatic function while maintaining shoulder analgesia, the latter by moving the injection site farther from the phrenic nerve.

More recently, the field has moved beyond the concept of selective neural blockade alone. Combined distal approaches, including infraclavicular–anterior suprascapular strategies, have been proposed to achieve comprehensive sensory coverage through the coordinated blockade of multiple neural pathways while minimizing proximal spread within the interscalene compartment [44]. Emerging evidence has also explored costoclavicular and other distal brachial plexus approaches as potential diaphragm-sparing alternatives, reflecting a broader trend toward individualized regional anesthesia strategies tailored to patient-specific respiratory and functional requirements [37]. Contemporary reviews increasingly describe shoulder regional anesthesia as a continuum of techniques that differ in their balance between analgesic efficacy, motor preservation, respiratory safety, and technical complexity rather than as a hierarchy dominated by a single reference standard [47,48].

Within this evolving framework, SOFPB occupies a distinctive position. Because it relies on interfascial spread toward multiple structures involved in shoulder sensation [10,13] rather than on blockade of a single discrete nerve, SOFPB may represent a conceptual bridge between selective nerve blocks and fascial plane blocks, combining anatomical selectivity with the broader distribution characteristic of interfascial approaches.

This distinction may have important clinical implications. Contemporary shoulder anesthesia increasingly seeks to optimize not only pain control but also respiratory function, motor preservation, and postoperative recovery. In this context, the theoretical value of SOFPB lies in its potential to influence several sensory pathways through a single injection while limiting direct exposure of the phrenic nerve to local anesthetic. Although preliminary clinical findings and anatomical investigations support this possibility, the available evidence remains considerably less mature than that supporting ASSB or STB [10,11,44].

### 5.2. Potential Advantages, Risks, and Current Clinical Position of SOFPB

Among the growing number of diaphragm-sparing shoulder regional anesthesia techniques, SOFPB has several hypothesized anatomical and practical advantages. These potential advantages must be balanced against equally important limitations, including uncertain injectate spread, non-standardized nomenclature, unquantified diaphragmatic risk, and limited comparative clinical evidence.

Among the growing number of diaphragm-sparing shoulder regional anesthesia techniques, SOFPB possesses several distinctive anatomical and conceptual features that may differentiate it from conventional neural blockade strategies. Although the current evidence base remains limited, available anatomical and clinical data suggest several potential advantages worthy of further investigation.

First, SOFPB is designed to avoid direct injection within the interscalene compartment, thereby potentially reducing phrenic nerve exposure. Respiratory preservation has become a major objective in contemporary shoulder regional anesthesia, particularly in patients with obesity, chronic pulmonary disease, advanced age, or limited respiratory reserve [42,49]. Unlike ISB, which frequently results in ipsilateral hemidiaphragmatic paresis, SOFPB targets the sub-omohyoid fascial compartment and may, therefore, reduce the likelihood of direct phrenic nerve blockade. This concept is consistent with the broader movement toward diaphragm-sparing regional anesthesia techniques that seek to preserve respiratory function while maintaining clinically effective analgesia [7,18,19,20,21,25,28,29,30].

Third, SOFPB may occupy an intermediate position between proximal and distal diaphragm-sparing approaches. Recent studies of superior trunk block, costoclavicular block, and related techniques have demonstrated that respiratory outcomes are strongly influenced by the anatomical location of injection and the extent of local anesthetic spread [36,37,50,51,52]. Whereas highly proximal blocks risk phrenic nerve involvement and more distal approaches may provide less predictable shoulder coverage, SOFPB theoretically combines anatomical selectivity with broader interfascial distribution. At present, however, this balance remains a testable hypothesis rather than an established clinical advantage.

Third, SOFPB may occupy an intermediate position between proximal and distal diaphragm-sparing approaches. Recent studies of superior trunk block, costoclavicular block, and related techniques have demonstrated that respiratory outcomes are strongly influenced by the anatomical location of injection and the extent of local anesthetic spread [36,37,50,51,52]. Whereas highly proximal blocks risk phrenic nerve involvement and more distal approaches may provide less predictable shoulder coverage, SOFPB theoretically combines anatomical selectivity with broader interfascial distribution. This balance may represent a unique advantage if future studies confirm reliable analgesic efficacy with limited respiratory compromise.

Despite these theoretical advantages, most remain hypothesis-generating rather than proven. Existing evidence suggests that respiratory preservation depends not only on target location but also on injectate volume, spread characteristics, and patient-specific anatomy [34,35,50,51]. Potential risks include inadvertent spread toward the phrenic nerve, vascular puncture, incomplete analgesia due to insufficient fascial plane spread, and unintended brachial plexus motor involvement. Accordingly, the clinical value of SOFPB will ultimately depend on whether future comparative studies demonstrate meaningful benefits beyond those already achieved with established diaphragm-sparing techniques.

Despite these theoretical advantages, most remain hypothesis-generating rather than proven. Existing evidence suggests that respiratory preservation depends not only on target location but also on injectate volume, spread characteristics, and patient-specific anatomy [34,35,50,51]. Accordingly, the unique value of SOFPB will ultimately depend on whether future comparative studies demonstrate clinically meaningful benefits beyond those already achieved with established diaphragm-sparing techniques.

### 5.3. Remaining Uncertainties and Unanswered Questions

Despite increasing interest in SOFPB, the available evidence remains insufficient to define its mechanism, optimal application, and clinical role, even though its anatomical rationale is compelling.

The first uncertainty concerns the precise mechanism of analgesia. Cadaveric and anatomical investigations suggest that injectate deposited within the sub-omohyoid compartment may spread toward the suprascapular nerve, superior trunk region, and adjacent cervical fascial planes [10,13]. However, the relative contribution of each structure to the observed clinical effect remains unclear. It is, therefore, uncertain whether SOFPB should be regarded primarily as a suprascapular nerve block, an indirect superior trunk block, an interfascial plane block, or a combination of these mechanisms. Further anatomical validation using advanced imaging and injectate tracking studies is required to clarify the dominant pathways responsible for analgesia.

A second unresolved issue is the optimal injectate volume and concentration. Recent dose-finding investigations involving anterior suprascapular and superior trunk blocks have demonstrated that local anesthetic volume significantly influences both analgesic efficacy and respiratory outcomes [26,27,34,35,51]. Similar studies have not yet been performed for SOFPB. Consequently, the minimum effective volume, optimal concentration, and volume-dependent spread characteristics remain unknown. This knowledge gap is clinically relevant because excessive injectate spread may increase the likelihood of unintended phrenic nerve involvement, whereas insufficient spread may compromise analgesic effectiveness.

The true diaphragm-sparing profile of SOFPB also remains uncertain. Although the technique is anatomically positioned farther from the phrenic nerve than conventional interscalene blockade, cadaveric evidence suggests that spread toward phrenic nerve-related structures may still occur under certain conditions [13]. Moreover, studies evaluating alternative shoulder blocks have demonstrated substantial differences in respiratory outcomes depending on injection site, injectate volume, and assessment methodology [36,46,50,53]. Whether SOFPB consistently preserves diaphragmatic function better than established techniques such as ASSB or STB has not been determined.

Another important unanswered question concerns motor preservation and functional recovery. Contemporary shoulder regional anesthesia increasingly prioritizes postoperative function in addition to pain control. However, most published SOFPB studies have focused on analgesic outcomes, anatomical spread, or respiratory effects. Data regarding upper-extremity motor weakness, rehabilitation milestones, quality-of-recovery scores, and patient-reported functional outcomes are largely unavailable.

Finally, the comparative role of SOFPB remains undefined. Several diaphragm-sparing alternatives—including ASSB, STB, costoclavicular block, high-thoracic erector spinae plane block, and modified supraclavicular approaches—have been evaluated in randomized controlled trials and systematic reviews [7,18,19,20,21,25,36,37,38,52,54,55]. In contrast, direct head-to-head comparisons involving SOFPB remain scarce. As a result, it remains unclear whether SOFPB offers clinically meaningful advantages over existing diaphragm-sparing techniques or simply represents another variation within an expanding spectrum of anatomy-guided shoulder blocks.

**Figure 4:**
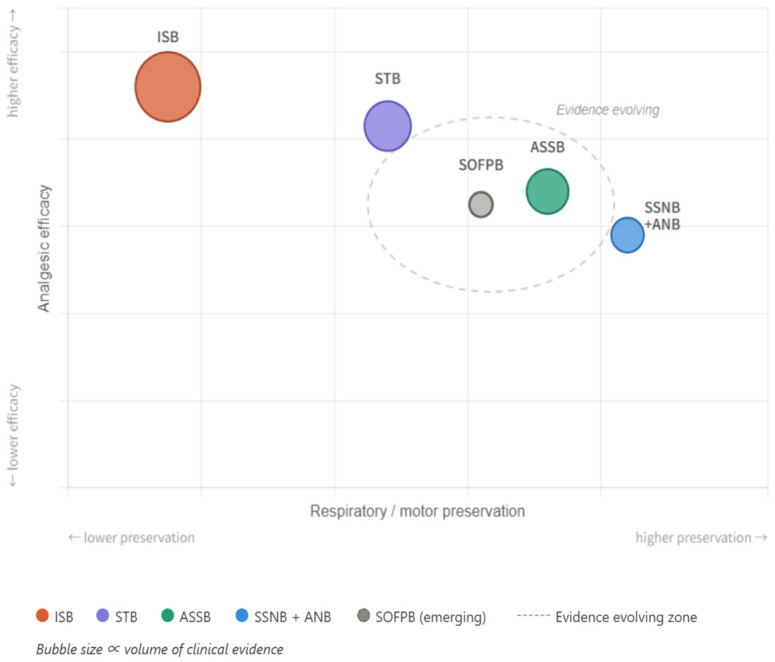
Proposed clinical role of SOFPB among shoulder regional anesthesia techniques.

Addressing these uncertainties will be essential before SOFPB can be fully integrated into routine clinical practice. The next phase of investigation should, therefore, focus not only on demonstrating efficacy but also on defining where SOFPB fits within the broader landscape of contemporary shoulder regional anesthesia (Figure 4, Table 5).

Future research should be prioritized in a stepwise manner. The first priority is anatomical validation and nomenclature standardization, the second is dose and spread optimization with objective respiratory assessment, and the third is adequately powered clinical comparison against established diaphragm-sparing alternatives. This sequence is necessary because comparative trials will be difficult to interpret unless the target plane, injectate volume, and outcome definitions are first standardized.

First, anatomical studies are needed to clarify the mechanisms underlying SOFPB and to standardize its technical definition. Future cadaveric and imaging-based investigations should characterize the consistency and extent of injectate spread within the sub-omohyoid compartment and determine its relationship to the suprascapular nerve, superior trunk, phrenic nerve, and adjacent cervical fascial planes [10,13,46]. Combining ultrasound-guided injection with magnetic resonance imaging or other advanced imaging techniques may improve understanding of the pathways responsible for analgesia and potential respiratory effects.

Second, dose-optimization studies are required. Recent investigations involving anterior suprascapular and superior trunk blocks have demonstrated that local anesthetic volume substantially influences both analgesic efficacy and the incidence of hemidiaphragmatic paresis [26,27,34,35,51]. Comparable studies have not yet been performed for SOFPB. Establishing the minimum effective volume and defining volume-dependent spread characteristics, ideally with standardized diaphragmatic ultrasound and pulmonary function assessment, will be essential for maximizing efficacy while minimizing unintended neural blockade.

Third, future clinical trials should directly compare SOFPB with established diaphragm-sparing alternatives. Although ASSB, STB, upper trunk block, costoclavicular block, and other selective approaches have been evaluated in randomized trials and systematic reviews, comparative data involving SOFPB remain limited [7,18,19,20,21,25,36,37,38,44,52,53,54,55]. Adequately powered multicenter trials incorporating standardized pain, opioid-related, respiratory, motor, and patient-centered outcomes are necessary to determine whether SOFPB offers clinically meaningful advantages over existing techniques.

Third, future clinical trials should directly compare SOFPB with established diaphragm-sparing alternatives. Although ASSB, STB, upper trunk block, costoclavicular block, and other selective approaches have been evaluated in randomized trials and systematic reviews, comparative data involving SOFPB remain limited [7,18,19,20,21,25,36,37,38,44,52,53,54,55]. Adequately powered multicenter trials incorporating standardized pain, respiratory, and functional outcomes are necessary to determine whether SOFPB offers clinically meaningful advantages over existing techniques.

Fourth, greater emphasis should be placed on patient-centered outcomes. Future investigations should evaluate upper-extremity motor function, rehabilitation milestones, quality-of-recovery measures, patient satisfaction, return to daily activities, and healthcare utilization. These outcomes may ultimately be more relevant than isolated pain scores when assessing the overall value of a regional anesthesia technique.

Finally, continuous catheter-based applications of SOFPB warrant exploration. Continuous suprascapular and superior trunk techniques have demonstrated the feasibility of prolonged shoulder analgesia while potentially reducing some limitations associated with interscalene blockade [56]. Whether a continuous SOFPB catheter can provide sustained analgesia while preserving respiratory and motor function remains unknown and represents an important area for future investigation.

Collectively, these research priorities may help establish whether SOFPB should be regarded as a complementary option within the expanding spectrum of diaphragm-sparing shoulder blocks or as a distinct technique with unique clinical advantages.

The sub-omohyoid fascial plane block (SOFPB) is an emerging interfascial concept within the expanding spectrum of diaphragm-sparing regional anesthesia techniques for shoulder surgery. In the present review, SOFPB is best understood as an operational umbrella term for related sub-omohyoid approaches that deposit local anesthetic deep to the omohyoid muscle near the superior trunk–suprascapular nerve complex, rather than as a fully standardized single block.

Current evidence suggests anatomical plausibility and preliminary clinical feasibility, with potential analgesic effects mediated by suprascapular nerve involvement, partial superior trunk coverage, and variable interfascial spread. However, the mechanistic basis, optimal injectate volume, consistency of spread, true diaphragm-sparing profile, motor-sparing characteristics, and comparative effectiveness remain uncertain. Much of the supporting evidence is indirect, derived from related sub-omohyoid techniques, cadaveric dye studies, or small clinical reports, and should not be interpreted as proof that SOFPB is equivalent or superior to established alternatives such as anterior suprascapular nerve block or superior trunk block.

At present, SOFPB should be considered a promising but unvalidated adjunct within individualized shoulder analgesia rather than a replacement for established regional anesthesia techniques. Future work should prioritize standardized nomenclature and technique description, imaging-confirmed spread analysis, dose-finding studies with objective diaphragmatic assessment, and head-to-head randomized trials incorporating pain, respiratory, motor, and patient-centered outcomes.

At present, SOFPB should be regarded as an evolving and promising technique rather than a proven replacement for existing shoulder blocks. Future anatomical investigations, dose-finding studies, and randomized comparative trials are needed to clarify its role within contemporary shoulder regional anesthesia. As interest in diaphragm-sparing and patient-centered regional anesthesia continues to grow, SOFPB represents a potentially valuable addition to the anesthesiologist’s armamentarium and warrants further rigorous evaluation.

## Figures and Tables

**Table 1:** Terminology, anatomical scope, and technical considerations for sub-omohyoid approaches included within the SOFPB framework.

Evidence Category and Key Interpretation	Anatomical Definition and Relationship to SOFPB	Reported Term/Study
Cadaveric indirect evidence. SSN and superior trunk staining support anatomical plausibility, but phrenic staining indicates possible unintended spread.	SSN beneath the inferior belly of the omohyoid; dye injected deep to the omohyoid. Anatomically related to the SOFPB framework.	Subomohyoid suprascapular nerve block—Sehmbi et al. (2019) [9]
Multicenter RCT; related clinical evidence. Supports analgesic feasibility, but conclusions apply to that specific technique.	Anterior SSN origin/superior trunk region using a sub-omohyoid anterior approach. Closest related clinical technique but not identical to a standardized SOFPB.	Subomohyoid anterior suprascapular block—Abdallah et al. (2020) [10]
RCT; related clinical evidence. Reported markedly lower phrenic nerve block than low-volume ISB with similar analgesic outcomes.	Distal subomohyoid/infraclavicular region with 25 mL ropivacaine 0.5%. Related diaphragm-sparing sub-omohyoid approach.	Infraclavicular subomohyoid block—Taha et al. (2019) [11]
Case report; very low-level evidence. Demonstrates feasibility of prolonged analgesia only.	Catheter-based subomohyoid SSN pathway. Continuous variant of a related sub-omohyoid approach.	Continuous subomohyoid suprascapular nerve block—Dhir et al. (2021) [12]
Cadaveric indirect safety/mechanistic evidence. Demonstrated dorsal dye spread toward phrenic nerve-related structures in some specimens.	Proximal SSN at the infra-omohyoid level; cadaveric dye injection. Mechanistically relevant, not a clinical SOFPB trial.	Anterior suprascapular approach with phrenic pathway assessment—Diwan et al. (2025) [13]
Conceptual synthesis; not an independent evidence level. Integrates related data while acknowledging nomenclature and mechanistic uncertainty.	Interfascial compartment deep to the omohyoid near the superior trunk–SSN complex. Used as an operational umbrella term requiring standardization.	SOFPB as used in this review

**Table 2:** Major neural pathways involved in shoulder pain and their potential relevance to SOFPB.

Nerve	Origin	Principal Sensory Territory	Clinical Relevance for Shoulder Analgesia	Potential Relevance to SOFPB
Suprascapular nerve	Superior trunk (C5–C6)	Posterior and superior glenohumeral capsule, acromioclavicular joint	Major contributor to shoulder joint sensation; common target for shoulder blocks	High
Axillary nerve	Posterior cord (C5–C6)	Inferior and posterolateral glenohumeral capsule, deltoid region	Important contributor to postoperative shoulder pain, particularly after arthroscopic procedures	Moderate
Lateral pectoral nerve	Lateral cord (C5–C7)	Anterosuperior capsule and surrounding soft tissues	One of the “three bridges” contributing to shoulder nociception	Moderate
Upper subscapular nerve	Posterior cord (C5–C6)	Anterior capsule and subscapularis region	Secondary articular contribution	Low–Moderate
Lower subscapular nerve	Posterior cord (C5–C6)	Inferior capsule and periarticular structures	Secondary sensory contribution	Low–Moderate
Supraclavicular nerves	Cervical plexus (C3–C4)	Skin over clavicle, acromion, superior shoulder	Important for incision and portal-site pain	Variable
Articular branches of cervical plexus *	Cervical plexus	Variable contribution to clavicular and periosteal sensation	May explain incomplete analgesia with isolated brachial plexus blockade	Variable

* Anatomical variability has been reported in cadaveric studies.

**Table 3:** Clinical and anatomical evidence evaluating sub-omohyoid plane block and related sub-omohyoid approaches for shoulder analgesia, stratified by evidence type and directness.

Interpretive Weight for SOFPB	Quantitative/Effect-Size Summary	Intervention and Comparator	Study/Evidence Level
Conceptual rationale only; no efficacy or safety inference.	No comparative effect size; feasibility concept only.	Subscapularis and sub-omohyoid plane blocks/none	Sondekoppam et al. (2016) [24]—concept report
Clinically relevant but related, not identical, technique.	Phrenic nerve block: 5.6% vs. 88.9% (*p* < 0.001); no significant difference in analgesia duration, 24-h morphine use, or satisfaction.	Infraclavicular subomohyoid block vs low-volume ISB in 72 shoulder arthroscopy patients.	Taha et al. (2019) [11]—randomized blinded study
Strongest related clinical evidence; specific to subomohyoid anterior suprascapular block.	24-h rest-pain AUC: 13.2 ± 2.0 vs. 12.8 ± 1.4 U; mean difference (ISB minus suprascapular) −0.3 U (90% CI, −0.8 to 0.2), above the −4.4 U noninferiority margin. Superior trunk sensory-motor block: 69/69 vs. 65/67; RR 0.97 (90% CI, 0.93–1.01).	Subomohyoid anterior suprascapular block vs. ISB; 136 analyzed patients.	Abdallah et al. (2020) [10]—multicenter double-blind noninferiority RCT
Feasibility only; cannot establish comparative efficacy or safety.	No comparative effect size; successful continuous analgesia reported as part of multimodal management.	Continuous bilateral subomohyoid suprascapular catheters/none.	Dhir et al. (2021) [12]—case report
Indirect anatomical plausibility only; not clinical outcome evidence.	SSN staining 90%; superior trunk 90%; middle trunk 80%; inferior trunk 20%; mild phrenic staining 20%.	Subomohyoid suprascapular block; 10 injections in five fresh cadavers.	Sehmbi et al. (2019) [9]—cadaveric dye study
Cautionary indirect mechanistic evidence; does not quantify clinical HDP risk.	Phrenic nerve stained 41.7%; SSN and posterior division stained 100%; inferior trunk unstained 100%; anterior division of superior trunk observed in 75%.	Anterior suprascapular approach at infra-omohyoid level; 12 neck specimens.	Diwan et al. (2025) [13]—cadaveric dye study

**Table 4:** Comparison of SOFPB with established diaphragm-sparing regional anesthesia techniques for shoulder surgery. Risk and motor descriptors are qualitative and reflect the comparative literature cited in this review; SOFPB entries are provisional given the limited direct evidence.

Technique	Injection Site/Target	Hemidiaphragmatic Paresis Risk	Upper-Limb Motor Effect	Analgesic Evidence (vs. ISB)	Key References
Interscalene block (ISB)	Brachial plexus roots/trunks in the interscalene groove (C5–C6)	High (near-universal)	Frequent (dense brachial plexus block)	Reference standard; consistently effective analgesia	[4,6,23]
Anterior suprascapular nerve block (ASSB)	Suprascapular nerve at the sub-omohyoid/anterior cervical region	Low; reduced vs. ISB	Largely preserved	RCTs and meta-analyses: non-inferior analgesia with better diaphragm preservation	[10,18,19,20,21,25,31,32,33]
Superior/upper trunk block (STB)	Superior (upper) trunk, distal to the roots (C5–C6)	Low; markedly reduced vs. ISB	Largely preserved	RCTs: non-inferior analgesia, phrenic-sparing	[4,7,28,29,30,34,35,36]
Costoclavicular block	Cords of the brachial plexus at the costoclavicular space (infraclavicular)	Low (distal approach)	Variable; arm block possible	Emerging RCT evidence as a diaphragm-sparing option	[37,38]
Sub-omohyoid fascial plane block (SOFPB)	Interfascial sub-omohyoid compartment near the superior trunk–suprascapular nerve complex	Anticipated low (distal, interfascial); not yet well quantified	Anticipated preservation; limited direct data	Limited; feasibility and preliminary efficacy from related sub-omohyoid approaches	[9,10,11,12,13,24]

**Table 5:** Knowledge gaps and future research priorities for sub-omohyoid fascial plane block (SOFPB).

Domain	Current Evidence	Key Knowledge Gap	Future Research Priority
Anatomical mechanism	Cadaveric and anatomical studies suggest spread toward the suprascapular nerve, superior trunk region, and adjacent cervical fascial planes.	The dominant analgesic pathway remains unclear.	Cadaveric validation combined with MRI or advanced imaging studies to characterize injectate spread patterns.
Injectate volume optimization	Preliminary evidence from ASSB and STB indicates that injectate volume strongly influences efficacy and respiratory outcomes.	Minimum effective volume and volume-dependent spread characteristics for SOFPB are unknown.	Prospective dose-finding and volume-response studies.
Analgesic efficacy	Early clinical reports suggest favorable analgesic outcomes.	Comparative effectiveness relative to ASSB, STB, and other diaphragm-sparing techniques remains uncertain.	Multicenter randomized controlled trials with standardized pain and opioid-related outcomes.
Respiratory preservation	SOFPB is anatomically positioned farther from the phrenic nerve than ISB and may reduce phrenic nerve involvement.	The true incidence of hemidiaphragmatic paresis following SOFPB is unknown.	Studies incorporating diaphragmatic ultrasound, pulmonary function testing, and respiratory outcome assessment.
Motor preservation	Theoretical motor-sparing characteristics have been proposed.	Effects on upper-extremity motor function and rehabilitation are poorly defined.	Prospective evaluation of motor strength, functional recovery, and return-to-activity metrics.
Patient-centered outcomes	Existing studies focus primarily on pain and anatomical spread.	Quality of recovery, patient satisfaction, and long-term functional outcomes remain underreported.	Inclusion of validated patient-reported outcome measures (PROMs) and quality-of-recovery scores.
Comparative effectiveness	Several diaphragm-sparing alternatives (ASSB, STB, costoclavicular block) have established clinical evidence.	The relative position of SOFPB within the contemporary shoulder block armamentarium remains undefined.	Head-to-head comparative trials against established diaphragm-sparing techniques.
Continuous catheter applications	Evidence is extremely limited.	Feasibility, safety, and efficacy of continuous SOFPB are unknown.	Development and evaluation of continuous SOFPB catheter techniques for prolonged postoperative analgesia.
Standardization and nomenclature	Variability exists in terminology and technical descriptions across studies.	Lack of uniform definitions limits comparison between studies.	Consensus recommendations regarding nomenclature, block technique, and outcome reporting.

## Data Availability

No new data were created or analyzed in this study. Data sharing is not applicable to this article.
